# “Prophylaxis” and “Pyorrhoea Alveolaris”

**Published:** 1905-06-15

**Authors:** C. M. Wright

**Affiliations:** Cincinnati, Ohio


### "PROPHYLAXIS" AND "PYORRHOEA ALVEOLARIS."

BY C. M. WRIGHT, D.D.S., CINCINNATI, OHIO.

The term "prophylaxis" has acquired a distinct definition in dentistry. Here it means special *manipulative* treatment of *healthy tissues* to prevent the occurrence or recurrence of a definite disease.

Sixty to eighty percent of the teeth of mankind are lost by this disease in middle life and old age. Are there any special manipulations of these tissues which will prevent this loss? Successful prophylaxis must depend upon correct estimates of indirect as well as of direct causes. We are giving increased attention every day to operative measures for the thorough cleaning of teeth and roots as prophylactic in relation to the direct causes or local irritants.

The most conspicuous indirect cause of the early loss of teeth is non-use of the organ of mastication, which is built up of unique "transitory structures." (Talbot.)

Metchnikoff might say there is a "lack of harmony" between these structures evolved for and by ancient needs and their present uses and environment. This "lack" induces degenerations in form and substance, and places the unused organ in a menacing attitude toward the rest of the organism. With these views clearly before us, what shall our prophylaxis be?

*First.* To regulate the positions and occlusions of the teeth when necessary, so that we may have a perfect machine for mastication. This requires the exercise of a high degree of mechanical and surgical skill. Great advance in methods has been made in recent years in this department of dentistry.

*Second.* To cure individual teeth of their diseases and restore their forms by fillings, so that each may become a useful part of a concrete apparatus. This has occupied the efforts of the profession for years, and is still the most prominent feature of dental practice.

*Third.* When we have a perfect machine—whether the perfection is the result of natural growth and inheritance, or of tedious operative measures—we must provide *function.* We have not done this, but we must do it, if we are to be considered consistent and logical.

The functions of the human teeth are to gnaw bones, to grind grain, to chew twigs, to crack nuts and to perform the same offices that the same structures do in the mouths of the dog, the cat, the ox and the ass.

Altruistic table manners and modern cooking of foods do not permit of this usage, therefore we, the dentists, in the continuance of our prophlyactic practice should furnish artificial means of exercise, as substitutes for the functions which nature demands for the persisting health of these tissues.

This point having been neglected in general dental practice, we find no ingenious inventions and appliances for this work as we do in orthodontia and operative dentistry. We find not even a suggestion of an *electric tooth-percussor, or electric roller for gum massage.* We have no appliances for artificial exercises, which will take the place of grinding and biting. The necessity or instinct, we might say, for these exercises can be observed in the pet puppies that play about our hearths. How they will grind and gnaw a cinder or a bone for hours with their baby teeth! We suppress this instinct in the house-bred dog, as we do in our children, and the results in both cases are imperfectly developed structures and depreciated general health.

It is easy to believe that force—such as electricity, compressed air, or hand intelligently directed by the dentist to the teeth and gums and jaws of children, youths and adults, and systematically and persistently applied—would accomplish marvelous results in the structural development of these organs in early life, and in the reduction of the percentage of loss of teeth in middle life, from pyorrhea alveolaris.

Rhinologists recognize that the unused jaws, teeth, glands and muscles of mastication play an important role in the etiology of adenoids and malformations of the upper respiratory tracts; also in diseases of sinuses, septa and mucous membranes of the throat and nose. Vigorous and prolonged mastication and its accompanying insalivation of food before deglutition, bears a close and important relation to the nutrition of the body, also the septic condition of the lower bowel, with its ever-present threat of auto-infections from bacterial poisons, would be diminished, if fragments of unground and undigested foodstuffs were not permitted to enter. A treatise could be written on this part of the subject, but these are not the points which I wish to emphasize at this time. Rather that the dentist's responsibility should not cease when he has by his present high-class skill placed the teeth in perfect order orthopædically and dentally. He should provide artificial means for attaining the benefits of a natural function, and combat the degenerations resulting from unavoidable modern habits and environments. This is prophylaxis in dentistry, and its practice will open a new and very large field for earnest laborers.

The subject is so new, as far as practice is involved, that when we look over the field we find only some crude massage brushes for the gums, a dental chewing-gum recommended by a few dentists, and Dr. D. D. Smith's frequent polishing of the surfaces of the teeth with orange-wood sticks and pumice. With the possible faint suggestions buried in the chewing-gum habit of benefit to the masticatory apparatus, all of these are only proposed measures to prevent the action of *direct* causes. True prophylaxis should begin with mechanical efforts to hinder atrophic degenerations of muscles, glands and maxillæ in infancy and childhood, or during the period of active tissue growth and development. The idea being to assist in the development of normal arches for the teeth, thickened alveolar processes and firm gum tissue when these structures are developing. If thumb-sucking can cause malformation of the mouth during the period of growth, properly directed force can help toward perfect formation. Arches can be widened, muscles can be strengthened, osseous tissue can be developed and "occlusions" (Angle) can be directed by mechanical measures in early life, and function can be maintained during adult life, when we, the dentists, bring our ingenuity to bear on this wider prophylaxis.

The Kingsleys, Angles, Davenports, Cases, *et al.*, of the future, will show to us "function appliances" as ingenious and valuable as are the orthodontia devices of to-day. For infancy and childhood the hand alone may be employed in systems of massage, consisting of pressing, pushing, kneading and rubbing in the right directions on gums and palate, and of the muscles attaching the mandible. For youth and adult life, simple but effective incline planes might be arranged to operate by biting, and constructed of elastic materials with the direction of force skillfully arranged to simulate the natural grinding and biting effects as in mastication. Such treatment of healthy tissues would accomplish much in counteracting modern tendencies of civilization of the structural development of these organs the care of which in our day demands the highest culture of a distinct profession. With this prophylaxis, supplemented by Dr. D. D. Smith's *one* monumental idea—*frequency* of local scouring—we could prevent the loss of the teeth in middle life and old age. We shall have assisted nature in developing a perfect apparatus, and fortified the perfect tissues for a high degree of physiological resistance.—*Brief.*

